# Evaluation of Various *Escherichia coli* Strains for Enhanced Lycopene Production

**DOI:** 10.4014/jmb.2302.02003

**Published:** 2023-04-17

**Authors:** Jun Ren, Junhao Shen, Thi Duc Thai, Min-gyun Kim, Seung Ho Lee, Wonseop Lim, Dokyun Na

**Affiliations:** Department of Biomedical Engineering, Chung-Ang University, Seoul 06974, Republic of Korea

**Keywords:** Lycopene, strain selection, *Escherichia coli*, MG1655, metabolic engineering

## Abstract

Lycopene is a carotenoid widely used as a food and feed supplement due to its antioxidant, anti-inflammatory, and anti-cancer functions. Various metabolic engineering strategies have been implemented for high lycopene production in *Escherichia coli*, and for this purpose it was essential to select and develop an *E. coli* strain with the highest potency. In this study, we evaluated 16 *E. coli* strains to determine the best lycopene production host by introducing a lycopene biosynthetic pathway (*crtE*, *crtB*, and *crtI* genes cloned from *Deinococcus wulumuqiensis* R12 and *dxs*, *dxr*, *ispA*, and *idi* genes cloned from *E. coli*). The 16 lycopene strain titers diverged from 0 to 0.141 g/l, with MG1655 demonstrating the highest titer (0.141 g/l), while the SURE and W strains expressed the lowest (0 g/l) in an LB medium. When a 2 × YTg medium replaced the MG1655 culture medium, the titer further escalated to 1.595 g/l. These results substantiate that strain selection is vital in metabolic engineering, and further, that MG1655 is a potent host for producing lycopene and other carotenoids with the same lycopene biosynthetic pathway.

## Introduction

Lycopene in the carotenoid family is a widely used food and feed antioxidant supplement, as it is one of the most potent quenchers of singlet oxygen molecules [1, 2]. Moreover, the cosmetic and pharmaceutical industries utilize lycopene due to its antioxidant, anti-inflammatory, and anti-cancer properties [3-5]. Conventionally, lycopene is obtained through extraction from fruit, chemical synthesis, and microbial fermentation. In nature, many fruits such as tomato, guava, watermelon, and papaya contain lycopene in amounts as high as 0.3-1.4 ng/g [6-8]. However, fruit extracts cannot satisfy the large market demand for lycopene due to the unstable and limited supply of natural fruits and their low lycopene content [9, 10]. Although chemical synthesis may be an alternative method, it is unappealing due to low yield, high cost, and poor quality [9, 11]. Therefore, lycopene production through microbial fermentation has recently become a promising strategy as it enables stable lycopene production through sustainable processes [12, 13].

**Fig. 1:**
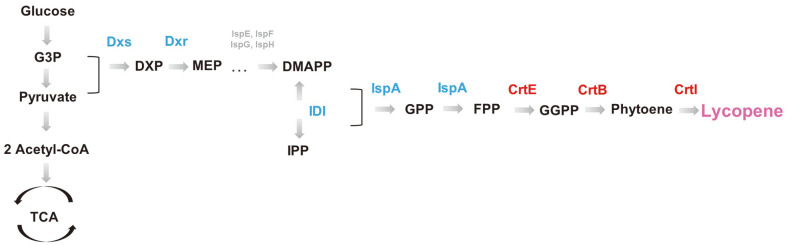
Lycopene biosynthetic pathway. The inherent *E. coli* metabolic pathway can only synthesize the lycopene precursor farnesyl diphosphate (FPP) in lycopene synthesis. Inherent *E. coli* genes (blue) were additionally expressed to enhance internal metabolic flux towards FPP. The red-colored genes derived from *Deinococcus wulumuqiensis* R12 were also introduced to produce lycopene from FPP. G3P, glyceraldehyde 3-phosphate; DXP, 1-deoxy-d-xylulose-5-phosphate; MEP, methylerythritol phosphate; DMAPP, dimethylallyl diphosphate; IPP, isopentenyl diphosphate; GPP, geranyl diphosphate; GGPP, geranylgeranyl diphosphate; *dxs*, 1-deoxy-*D*-xylulose 5-phosphate synthase; *dxr*, 1-deoxy-*D*-xylulose 5-phosphate reductoisomerase; *idi*, isopentenyl diphosphate isomerase; *ispA*, encoding farnesyl diphosphate synthase (*ispA*); *crtE*, geranylgeranyl diphosphate synthase; *crtB*, phytoene synthase; *crtI*, phytoene desaturase.

Many recent attempts have been made to produce lycopene from metabolically engineered prokaryotic cells [14-19]. Lycopene is a C_40_ carotenoid pigment synthesized from isopentenyl pyrophosphate (IPP) and dimethylallyl diphosphate (DMADP) (Fig. 1) [20-22]. In *Escherichia coli*, the 2-*C*-methyl-*D*-erythritol 4-phosphate (MEP) pathway produces these two precursors. *E. coli* harbors enzyme genes to convert the two precursors to farnesyl diphosphate (FPP) while heterologous geranylgeranyl diphosphate synthase (*crtE*), phytoene synthase (*crtB*), and phytoene desaturase (*crtI*) expressions allow the conversion of FPP to lycopene [23-25]. However, the MEP pathway in *E. coli* is not active enough to drive high lycopene production. Therefore, we overexpressed four prominent intermediate enzyme genes, 1-deoxy-*D*-xylulose-5-phosphate synthase (*dxs*) [26], 1-deoxy-*D*-xylulose 5-phosphate reductoisomerase (*dxr*), isopentenyl diphosphate isomerase (*idi*) [27], and farnesyl diphosphate synthase (*ispA*) [28], to drive metabolic flux towards IPP and DMAPP and enhance the lycopene biosynthetic pathway.

Among bacterial species, *E. coli* is widely utilized as a base microorganism in lycopene metabolic engineering due to its relatively fast growth and various associated, well-established microbial engineering techniques. *E. coli* comprises diverse strains exhibiting different physiological properties and metabolic activities [29]; however, only certain strains can be easily manipulated or accessed for metabolic engineering without considering strain-to-strain metabolic capacity differences. Consequently, there have been several efforts to produce lycopene via various engineering strategies. For example, overexpressing *dxs* in an *E. coli* DH5α flask culture supplied 22 mg/l of lycopene [26]; introducing the mevalonate pathway in an *E. coli* YBS125 flask culture provided 4 .28 mg/l [30]; fermenting a high cell density feed-batch in *E. coli* K12 produced 220 mg/l [31], and fermenting a large-scale fed-batch in *E. coli* L13 cultivated 1.23 g/l [17].

Previous efforts have focused on engineering strategies rather than identifying the best strain, which is our aim in the present study. First, we constructed plasmids for metabolic lycopene production in *E. coli* strains. Next, plasmids were introduced to 16 strains, and their lycopene production abilities were evaluated. Overall, *E. coli* MG1655 expressed the highest lycopene production capacity with a titer 10 to 16 times higher than commonly used laboratory strains, such as DH5α and BL21 (DE3).

## Materials and Methods

### DNA Manipulation and Plasmid Construction

The *Deinococcus wulumuqiensis* R12 *crtE*, *crtB*, and *crtI* genes were cloned through PCR into the pWA plasmid containing a ColE1 origin and the ampicillin resistance gene [32]. Six polycistronic gene *crtEBI* clusters (pWA-EBI, pWA-EIB, pWA-BEI, pWA-BIE, pWA-IEB, and pWA-IBE plasmids; Table 1) were constructed and expressed under synthetic promoter BBa_J23118 control obtained from the Anderson collection (http://parts.igem.org/Promoters/Catalog/Anderson). The *E. coli* DH5*α*-originated genes *dxs*, *dxr*, *ispA*, and *idi* were cloned into a plasmid harboring a replication p15A origin and a chloramphenicol resistance gene (pA-SRAI plasmid). These four genes were expressed under the P_BAD_ promoter regulated by AraC protein and arabinose. Table 1 organizes the primers and plasmids used in this study.

### Bacterial Strains and Media

The *E. coli* DH5α strain was used for cloning and plasmid preparation. *D. wulumuqiensis* R12 was purchased from the Korean Agricultural Culture Collection (KACC, http://www.genebank.go.kr/eng). Sixteen *E. coli* strains were evaluated for lycopene production: K-12 strains (DH5α, SURE, MG1655, JM110, XL10-Gold, XL1-Blue, LS5218, W3110, W3110ΔlacI, SM-10, TOP10, JM109, and NEB Turbo), B strain (BL21(DE3)), K-12 and B hybrid strain (HB101), and W strain. The K-12 strains are frequently used in laboratory and industrial processes, B strains primarily produce recombinant proteins, the W strain has a high growth rate and small by-product production, and the hybrid strain is often used for cloning experiments [33]. *D. wulumuqiensis* R12 was cultured in a polypeptone/yeast extract/magnesium medium (10 g/l of polypeptone, 2 g/l of yeast extract, 1 g/l of MgSO_4_·7H_2_O) at 37°C. All *E. coli* strains were cultured in an LB broth (1% tryptone, 0.5% yeast extract, and 1%NaCl) at 37°C with 25 μg/ml of chloramphenicol and 100 μg/ml of ampicillin). A 2 × YTg medium was used for lycopene production (16 g/l tryptone, 10 g/l yeast extract, 5 g/l NaCl, and 20% (w/v) of glycerol).

### HPLC Lycopene Measurement

A single metabolically engineered strain colony was incubated overnight in 5 ml of LB at 37°C and 230 rpm to measure lycopene production. Cells were inoculated into 50 ml of LB with 1% L-arabinose (Bio Basic, CAS#5328-37-0, Canada) and appropriate antibiotics, followed by incubation at 30°C and 200 rpm for 60 h. All experiments were performed in the dark because lycopene is light-sensitive, and lycopene measurements were repeated thrice. A Biotek Synergy H1 plate reader (Winooski, VT, USA) measured cell growth (OD_600_). At 48 and 60 h post-incubation, cells from 50 ml of culture broth were harvested through centrifugation at 7600 ×*g* and 4°C for 5 min. Cell pellets were washed once with distilled water, resuspended in acetone, and incubated at 55°C for 15 min. After centrifugation (7,600 ×*g*, 25°C, 10 min), HPLC was used to analyze the supernatant for lycopene quantification.

For lycopene analysis, 20 μl of a sample was analyzed by isocratic HPLC with a ZORBAX Eclipse Plus C18 column (4.6 × 150 mm, 5 μm; Agilent, USA) and a mobile phase composed of 80% acetone, 15% methanol, and 5%isopropanol at a constant flow rate of 1 ml/min for 20 min at 30°C. A commercially available lycopene (Sigma-Aldrich, USA) was used as a standard, and acetone-extracted lycopene was detected at 472 nm. All experiments were conducted under dark conditions to avoid lycopene isomerization by light [34, 35].

## Results

### Lycopene Biosynthetic Pathway Construction

**Fig. 2:**
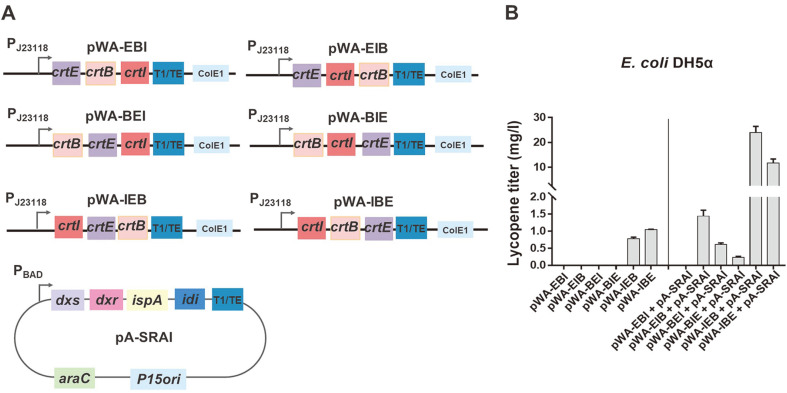
Effects of polycistronic *crtE*, *crtB*, and *crtI* gene clusters on lycopene production in E coli DH5α. (**A**) Constructed polycistronic *crtE*, *crtB*, and *crtI* gene clusters. (**B**) Lycopene titers produced from *crtE*, *crtB*, and *crtI* genes with or without plasmid pA-SRAI in *E. coli* DH5α within an LB medium for 48 h. All experiments were performed in the dark, and samples were prepared in triplicate. Error bars indicate standard deviations.

Fig. 1 illustrates the metabolic pathway toward lycopene. *E. coli* can inherently produce the lycopene precursor farnesyl diphosphate (FPP); however, it lacks the enzymes that convert FPP to lycopene. First, three *D. wulumuqiensis* R12-derived genes (*crtE*, *crtB*, and *crtI* genes) were cloned into a mid-copy plasmid containing a ColE origin (pWA plasmid) and transcribed by a synthetic constitutive promoter (BBa_J23118) for lycopene biosynthesis (Fig. 2). As the lycopene pathway consumes the intermediate metabolites in the glycolysis pathway, a mid-strength synthetic promoter (BBa_J23118) was employed to avoid cell growth diminution. Second, to drive more metabolic fluxes from the glycolysis pathway to FPP, the lycopene biosynthetic pathway precursor, four *E. coli* genes (*dxs*, *dxr*, *ispA*, and *idi*) were cloned into a mid-copy plasmid (pA-SRAI) and overexpressed under P_BAD_ promoter control. Like the lycopene pathway genes, a relatively weak promoter prevented growth defects.

### Polycistronic *crtE*, *crtB*, and *crtI* Gene Clusters on *E. coli* DH5α Lycopene Production

Six polycistronic gene clusters of the three genes were constructed and evaluated in *E. coli* DH5α with or without the plasmid pA-SRAI to investigate the lycopene biosynthetic gene (*crtE*, *crtB*, and *crtI*) order effect on lycopene titer (Fig. 2A). The six polycistronic gene clusters (pWA-EBI, pWA-EIB, pWA-BEI, pWA-BIE, pWA-IEB, and pWA-IBE; Table 1) were separately introduced into *E. coli* DH5α and the transformed cells were cultured in an LB medium for 48 h, as previously described [24]. The lycopene titer ranged from 0 to 1.0 mg/l without pA-SRAI internal flux enhancement (Fig. 2B). When the introduced pA-SRAI plasmid enhanced the internal metabolic flux towards FPP, the lycopene titer escalated to 24.1 mg/l (pWA-IEB). Since the *crtI*-*crtE*-*crtB* polycistronic gene cluster exhibited the highest lycopene titer with pA-SRAI co-expression, this gene cluster was selected for strain screening.

### Evaluating 16 *E. coli* Strains for Lycopene Production

Even strains of the same species may express eclectic production capabilities [29, 36, 37]. Thus, selecting the best strain is a critical step in metabolic engineering. Therefore, a selected polycistronic gene cluster (pWA-IEB) and internal flux-enhancing (pA-SRAI) plasmids were co-transformed into 16 *E. coli* strains to identify the best strain for lycopene production: 13 K-12 strains (DH5α, SURE, MG1655, JM110, XL10-Gold, XL1-Blue, LS5218, W3110, W3110ΔlacI, SM-10, TOP10, JM109, and NEB Turbo), one B strain (BL21(DE3)), one K-12 and B hybrid strains (HB101), and one W strain. Table 2 conveys the 16 *E. coli* strain genotypes. The transformed cells were cultured in an LB medium for 48 h. As we aimed to screen diverse strains in this study, no genomic engineering was conducted in the strains chosen. In addition, since the improper use of defined media could excessively reduce the lycopene titer past distinguishable strain capacities, we used LB and 2xYT as the high-nutrient media.

**Fig. 3:**
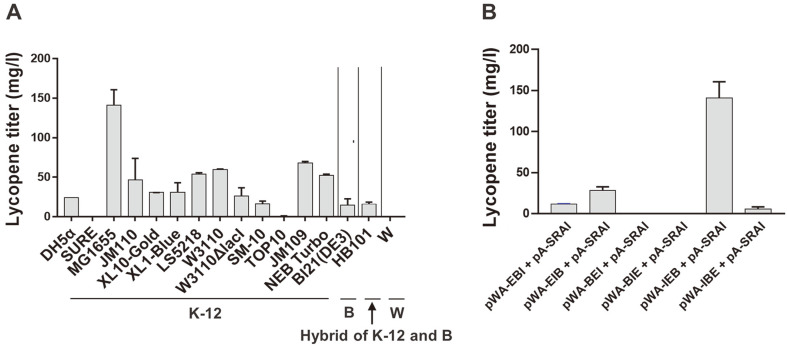
Evaluation of lycopene production using pWA-IEB and pA-SRAI in 16 *E. coli* strains. (**A**) Lycopene titers were produced from 16 *E. coli* strains with pWA-IEB and pA-SRAI. (**B**) Effects of the other polycistrons *crtE*, *crtB*, and *crtI* genes on lycopene titer in *E. coli* MG1655. Lycopene titers with *crtE*, *crtB*, and *crtI* (pWA-EBI to pWA-IEB) and *dxs*, *dxr*, *ispA*, and *idi* (pA-SRAI) genes were measured in *E. coli* MG1655 using an LB medium at 30°C and 200 rpm for 48 h. All experiments were performed in the dark, and samples were prepared in triplicate. Error bars indicate standard deviations.

Fig. 3A depicts the lycopene titers of the 16 strains. The MG1655 strain (141 mg/L) expressed the highest lycopene titer. Thus, the best strain for lycopene production was *E. coli* MG1655 with the *crtI*-*crtE*-*crtB* gene cluster and *dxs*, *dxr*, *ispA*, and *idi* gene expressions. The other five gene clusters were also evaluated to confirm whether the selected polycistronic gene cluster was the best in MG1655 (Fig. 3B). Consistent with the DH5α gene cluster evaluation results, the *crtI*-*crtE*-*crtB* cluster achieved the highest lycopene titer in MG1655. Currently, the most common *E. coli* strains for lycopene production are W3110, DH5α, XL1-Blue, and Bl21(DE3) [24, 26, 28]. However, these strains demonstrated a notably inferior lycopene production capability by comparison with MG1655, suggesting that the employment of MG1655 along with preexisting metabolic engineering strategies could substantially increase lycopene production.

### Lycopene Production Increase from a 2 × YTg Growth Enhancement Medium

**Fig. 4:**
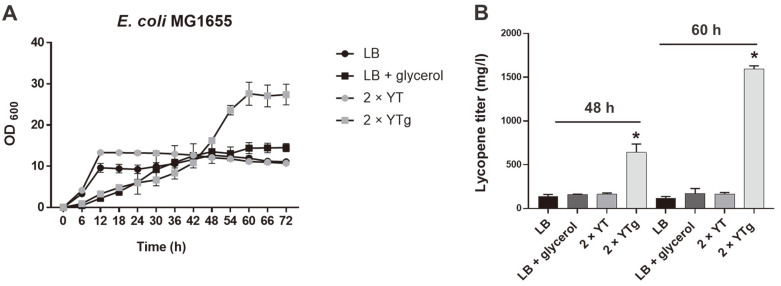
*E. coli* MG1655 strain evaluation with pWA-IEB and pA-SRAI in LB, LB (+ glycerol), 2×YT, or 2×YTg mediums. (**A**) *E. coli* MG1655 growth curves with pWA-IEB and pA-SRAI in LB, LB (+ glycerol), 2×YT, or 2×YTg. (**B**) Lycopene titers produced from the *E. coli* MG1655 stain with pWA-IEB and pA-SRAI in LB, LB (+ glycerol), 2×YTg, or 2×YTg after 48 or 60 h of incubation. All experiments were performed in the dark, and samples were prepared in triplicate. Asterisk (*) denotes *p*-value < 0.05. Error bars indicate standard deviations.

The metabolically engineered MG1655 strain reached a stationary phase at 12 h (Fig. 4A). We cultured cells in 2 × YT and 2 × YTg media to investigate if growth enhancement could further increase the lycopene titer. Glycerol is a viable carbon source for β-carotene and lycopene production [36, 38-42], as it increases glyceraldehyde 3-phosphate and pyruvate, which are imperative intermediates in central carbon metabolism extension to the MEP pathway [43, 44]. We determined that 2 × YTg significantly increased lycopene production due to the observed cell growth increase (Figs. 4A and 4B) [36, 42]. Interestingly, no significant lycopene titer or growth increase in LB, LB (+glycerol), and 2 × YT indicated that enriched nutrients and glycerol significantly increase cell growth and lycopene production. Furthermore, glycerol decreased cell growth rate during the initial phase, while the rich media (LB and 2 × YT) revealed an increase. However, cells incubated in the rich media reached a stationary phase sooner than cells grown with glycerol. This finding is potentially due to glycerol altering metabolism [45, 46], although this theory requires future study.

The engineered *E. coli* MG1655 strain was incubated in LB, LB (+ glycerol), 2 × YT, or 2 × YTg, and respective lycopene titers were measured at 48 and 60 h (Fig. 4B). Cell growth in LB, LB (+ glycerol), and 2 × YT did not increase after 12 or 48 h. Comparatively, cells in 2 × YTg reached a stationary phase at 60 h with a maximum OD 2.3, 1.9, and 2.2-fold higher than those grown in LB, LB (+ glycerol), and 2 × YT. Notably, the OD of cells grown in LB, LB (+glycerol), 2 × YT, and 2 × YTg was similar at 48 h (Fig. 4A), but cells grown in 2 × YTg achieved 4.6, 4.1, and 4-fold higher lycopene titers (651 mg/l). When the lycopene titer was measured at 60 h (stationary phase), the 2 × YTg titer was 1,595 mg/l, while those of LB, LB (+ glycerol), and 2 × YT cells were only 120, 171, and 166 mg/l, respectively. Our study confirms that nutrient enrichment and glycerol considerably promote lycopene production.

## Discussion

Selecting an optimal base strain is the first crucial step in metabolic engineering, as it could increase the lycopene titer from 0 mg/l (SURE) to 141 mg/l (MG1655). In this study we observed a substantial variety of lycopene titers in the 16 *E. coli* strains, even though they are derived from the same species. Consequently, the final lycopene titer achieved from the optimal *E. coli* strain MG1655, gene cluster, and medium combination was 1,595 mg/l, while the worst strains with non-optimal gene clusters did not express a detectable lycopene titer. To our knowledge, this titer was superior to the previous highest lycopene titer obtained from *E. coli* (1,240 mg/l) in a flask culture [17, 47], which emphasizes the value of strain selection. These results confirm that base strain selection is vital for enhanced substance production, and *E. coli* MG1655 is the optimal strain for lycopene metabolic engineering.

## Figures and Tables

**Table 1:** Plasmids and oligonucleotides within this study.

Plasmids	Description
pA-SRAI	*E. coli* *dxs*, *dxr*, *ispA*, and *idi* genes under P_BAD_ promoter control were cloned in a plasmid of p15A origin and a chloramphenicol resistance gene
pWA-EBI, EIB, BEI, BIE, IEB, IBE	*D. wulumuqiensis* R12-derived enzyme genes (*crtE* (E), *crtB* (B), *crtI* (I)) were cloned in various orders and transcribed under a synthetic promoter (BBa_J23118) control. The plasmid containing the three genes harbored ColE1 origin and an ampicillin resistance gene.
Primers	Oligonucleotide sequence^1^
crtE-F crtE-R	5'ATGCCTCGAGGAAGTGTACCGGAGAAGTGGC 3' 5'ATGCGGATCCATGCATGTCGACTATTTTTTCCTACTCGCATCCGC 3'
crtB-F crtB-R	5'ATGCCTCGAGTGAACGTGACGGAATTTTCGC 3' 5'ATGCGGATCCATGCATGTCGACGTGAACCTCTGAACATGTAGAAG 3'
crtI-F crtI-R	5'ATGCCTCGAGGCACCTTCTTCCCCTTTCTCTC 3' 5'ATGCGGATCCATGCATGTCGACCGTCCGTATGGGTTTTGGACAA 3'
Dxs-F Dxs-R	5'TAAAAGGAGACCCGGGATATGAGTTTTGATATTGCCAAATACCCGACCC 3' 5'CAGGGGCCTATTAATACTTATTGTTTATGCCAGCCAGGCCTTGATTTTGGCTTCC 3'
Dxr-F Dxr-R	5'AGTATTAATAGGCCCCTGATGAAGCAACTCACCATTCTGGGCTC 3' 5'GCGTTTTTTATTCCCTGACAGGGTTCAGCTTGCGAGACGCATCACCTCTTTTCTGGC 3'
ispA-F ispA-R	5'TCAGGGAATAAAAAACGCATGGACTTTCCGCAGCAACTCGAAGCCTGCG 3' 5'GCTGCCACTCCTGCTATACTCTTATTTATTACGCTGGATGATGTAGTCCGCTAGC 3'
Idi-F Idi-R	5'TATAGCAGGAGTGGCAGCATGCAAACGGAACACGTCATTTTATTGAATGC 3' 5'TTTGATGCCTGGCTCGAGTTATTTAAGCTGGGTAAATGCAGATAATCGTTTTC 3'

^1^Restriction enzyme site are underlined. XhoI (CTCGAG), BamHI (GGATCC)/SalI (GTCGAC)

**Table 2:** *E. coli* strains and their genotypes within this study.

*E. coli* strains		Genotype	Source or References
	DH5α	*F^–^ φ80lacZΔM15Δ(lacZYA-argF) U169 recA1 endA1 hsdR17(r_K_^–^, m_K_^+^) phoA supE44 λ^–^ thi-1 gyrA96 relA λ^–^*	Invitrogen
K-12 strains	SURE	*F? [proAB^+^ lacI^q^ lacZΔM15 Tn10(Tet^R^] endA1 glnV44 thi-1 gyrA96 relA1 lac recB recJ sbcC umuC::Tn5(Kan^R^ uvrC e14^–^(mcrA^–^) Δ(mcrCB-hsdSMR-mrr)171*	Stratagene
	MG1655	*F^–^ λ^–^ ilvG^–^ rfb-50 rph-1*	[48]
	JM110	*rpsL (Strr) thr leu thi-1 lacY galK galT ara tonA tsx dam dcm supE44 Δ(lac-proAB) [F´ traD36 proAB lacIqZΔM15]*	Stratagene
	XL10-Gold	*TetrD(mcrA)183 D(mcrCB-hsdSMR-mrr)173 endA1 supE44 thi-1 recA1 gyrA96 relA1 lac Hte [F´ proAB lacIqZDM15 Tn10 (Tetr) Amy Camr]*	Stratagene
	XL1-Blue	*recA1 endA1 gyrA96 thi-1 hsdR17 supE44 relA1 lac [F´ proAB lacIqZΔM15 Tn10 (Tetr)]*	Stratagene
	LS5218	*F^+^, fadR601, atoC512 (Const)*	[49]
	W3110	*K12 F- (rmD-rmE)*	[50]
	W3110ΔlacI	*K12F-(rmD-rmE) ΔlacI*	[50]
	SM-10	*thi thr leu tonA lacY supE recA::RP4-2-Tc::Mu *K*_m_ λpir*	[51]
	TOP10	*F_ mcrA D(mrr-hsdRMS-mcrBC) ¢80lacZD M15 DlacX74 recA1araD139 D(ara–leu)7697 galU galK rpsL (StrR) endA1 nupG*	Invitrogen
	JM109	*recA1, endA1, gyrA96, thi-1, hsdR17 (rkmk +), e14- (mcrA-), supE44, relA1, Δ (lac-proAB)/F’[traD36, proAB+, lacIq, lacZ Δ M15]*	TaKaRa
	NEB Turbo	*F' proABlacI^q^ ΔlacZM15 / fhuA2 Δ(lac-proAB) glnV galK16 galE15 R(zgb-210::Tn10)Tet^S^ endA1 thi-1 Δ(hsd)*	New England BioLabs
B strain	Bl21(DE3)	*F- ompT hsdSB (rB-mB-) gal dcm (DE3)*	Invitrogen
K-12 and B hybrid strain	HB101	*F– Δ(gpt-proA)62 leuB6 glnV44 ara-14 galK2 lacY1 Δ(mcrC-mrr) rpsL20 (Strr) xyl-5 mtl-1 recA13*	[52]
W strain	W	*ATCC 9637*	[53]
